# The (co-)occurrence of problematic video gaming, substance use, and psychosocial problems in adolescents

**DOI:** 10.1556/JBA.3.2014.013

**Published:** 2014-08-26

**Authors:** ANTONIUS J. VAN ROOIJ, DARIA J. KUSS, MARK D. GRIFFITHS, GILLIAN W. SHORTER, M. TIM SCHOENMAKERS, DIKE VAN DE MHEEN

**Affiliations:** ^1^IVO Addiction Research Institute, Rotterdam, The Netherlands; ^2^Erasmus MC, Rotterdam, The Netherlands; ^3^Birmingham City University, Birmingham, UK; ^4^International Gaming Research Unit, Psychology Division, Nottingham Trent University, Nottingham, UK; ^5^Bamford Centre for Mental Health and Wellbeing, University of Ulster, Londonderry, UK; ^6^MRC All-Ireland Hub for Trials Methodology Research, University of Ulster, Londonderry, UK; ^7^Department of Health Promotion, Maastricht University, Maastricht, The Netherlands

**Keywords:** problematic video gaming, Internet Gaming Disorder, online games, adolescents, alcohol, smoking, cannabis, loneliness, depression, negative self-esteem, social anxiety

## Abstract

*Aims:* The current study explored the nature of problematic (addictive) video gaming (PVG) and the association with game type, psychosocial health, and substance use. *Methods:* Data were collected using a paper and pencil survey in the classroom setting. Three samples were aggregated to achieve a total sample of 8478 unique adolescents. Scales included measures of game use, game type, the Video game Addiction Test (VAT), depressive mood, negative self-esteem, loneliness, social anxiety, education performance, and use of cannabis, alcohol and nicotine (smoking). *Results:* Findings confirmed problematic gaming is most common amongst adolescent gamers who play multiplayer online games. Boys (60%) were more likely to play online games than girls (14%) and problematic gamers were more likely to be boys (5%) than girls (1%). High problematic gamers showed higher scores on depressive mood, loneliness, social anxiety, negative self-esteem, and self-reported lower school performance. Nicotine, alcohol, and cannabis using boys were almost twice more likely to report high PVG than non-users. *Conclusions:* It appears that online gaming in general is not necessarily associated with problems. However, problematic gamers do seem to play online games more often, and a small subgroup of gamers – specifically boys – showed lower psychosocial functioning and lower grades. Moreover, associations with alcohol, nicotine, and cannabis use are found. It would appear that problematic gaming is an undesirable problem for a small subgroup of gamers. The findings encourage further exploration of the role of psychoactive substance use in problematic gaming.

## Introduction

### Problematic gaming and ‘game addiction’

Although the term ‘game addiction’ and its synonyms such as compulsive, excessive, and problematic use are regularly and interchangeably used (Kuss & Griffiths, 2012b), the clinical validity and necessity of a potential new ‘game addiction’ construct remains undetermined (Kardefelt-Winther, 2014). Nonetheless, a proposed diagnosis for *Internet Gaming Disorder* was included in the Appendix (Section 3) of the DSM-5 in order to stimulate further research into the topic (American Psychiatric Association, 2013; Petry & O’Brien, 2013). This diagnosis is phrased as a “[p]ersistent and recurrent use of the Internet to engage in games, often with other players, leading to clinically significant impairment or distress as indicated by five (or more) of the following [criteria] in a 12-month period” (American Psychiatric Association, 2013, p. 795).

Much of the current work on ‘game addiction’ was conducted using survey studies. While a variety of instruments exist, they tend to be derived from a mix of criteria used for ‘substance use disorder’ and ‘gambling disorder’ - the latter being the only behavioral addictive disorder in the DSM-5 (Griffiths, 2005; Lemmens, Valkenburg & Peter, 2009; Rehbein, Kleimann & Mößle, 2010; van Rooij, Schoenmakers, van den Eijnden, Vermulst & van de Mheen, 2012) . Using this approach, studies from the US, Norway, Germany and the Netherlands indicate that ‘game addiction’ is prevalent in 0.6% to 11.9% of adolescents (Gentile, 2009; King, Delfabbro & Griffiths, 2012; Mentzoni et al., 2011; Rehbein et al., 2010; van Rooij, Schoenmakers, Vermulst, van den Eijnden & van de Mheen, 2011). A summarizing review by Ferguson et al. concludes that prevalence estimates of around 3.1% are probably most accurate (Ferguson, Coulson & Barnett, 2011).

When asked about their game behavior, a significant proportion of gamers indicate having problems controlling their behavior. Given the problems with measurement, it is unknown to what extent these findings of potentially problematic gamers (Ferguson et al., 2011) in healthy populations and/or gamer samples translate to potential clinical cases of game addiction. There is reason for caution as clinically reported numbers in the Netherlands - 411 gamers in addiction care treatment (Wisselink, Kuijpers & Mol, 2013) - diverge from conservative Dutch adolescent population estimates of 1.5% to 2% (Lemmens et al., 2009; van Rooij et al., 2011). Diagnosis remains difficult when there is little consensus in diagnostic criteria. Although suggestions for new scales have been made (Petry et al., 2014), these are currently non-validated and derive from internal ‘voting’ procedures. Meanwhile, validated measures do not fully correspond to the current DSM-5 criteria (King, Haagsma, Delfabbro, Gradisar & Griffiths, 2013).

The authors are cautious in the use of addiction terminology in survey research at a young age. Thus, we refer to problematic (video) gaming (PVG) in the current survey-based study of a healthy population sample. PVG is defined as an addictive-like behavior that includes experiencing: (a) a loss of control over the behavior, (b) conflicts with the self and with others, (c) preoccupation with gaming, (d) the utilization of games for purposes of coping/mood modification, and (e) withdrawal symptoms (van Rooij, 2011; van Rooij et al., 2012). This measurement approach (see ‘Methods’) places PVG on a dimensional continuum (Helzer, van den Brink & Guth, 2006) and includes the main dimensions of internet/game addiction (Lortie & Guitton, 2013) . Early adolescence is the specific focus in this study. This is a crucial period in development, an age-group that rapidly adopts (gaming) technology, and a demographic group that is frequently referred to in (clinical) reports about gaming (Gross, Juvonen & Gable, 2002; Subrahmanyam, Greenfield, Kraut & Gross, 2001).

### The association between online games and problematic gaming

PVG is most often associated with online multiplayer games (Council on Science and Public Health, 2007; van Rooij, Schoenmakers, van den Eijnden & van de Mheen, 2010). A German study (N =7761, only boys) found players classed as ‘dependent’ (three standard deviations or higher above the average for their computer game dependency scale KFN-CSAS-II) spent most of their game time playing online games. While these findings fit with the DSM-5 proposal for ‘internet gaming disorder’, the potential for addiction in offline and casual (smartphone) games is understudied in the literature (Rehbein & Mößle, 2013). Although the mechanisms that cause addictive gaming are unknown, authors have speculated about the role of reward features, the social nature of online games, endlessness (King et al., 2012), and satisfaction of various play motivations (Kuss, Louws & Wiers, 2012). The following hypotheses canbe formulated:

Hypothesis (1): Online gamers are thought to be most susceptible to problematic (addictive) video gaming, in comparison to those that play offline and casual games.

Hypothesis (2): Problematic gamers are thought to spend most oftheir time on online games, in comparison to those that play offline and casual games.

### Psychosocial health and school performance

Researchers have consistently found relationships between measures of PVG and psychosocial problems (e.g. Ko, Yen, Chen, Chen & Yen, 2005; Ng & Wiemer-Hastings, 2005; Rehbein et al., 2010; van Rooij et al., 2011; Wood, Gupta, Derevensky & Griffiths, 2004). Poor school performance has also been associated with PVG. While the relationships between PVG and decreased psychosocial health are evident, their interpretation is not. Some authors argue that PVG might better be viewed as a manifestation of an underlying issue such as depressive mood or loneliness (e.g. Wood, 2007). With this in mind, commonly associated psychosocial state characteristics are explored: depressive mood (Han & Renshaw, 2011; Mentzoni et al., 2011), loneliness (Caplan, Williams & Yee, 2009; van Rooij, Schoen-makers, van den Eijnden, Vermulst & van de Mheen, 2013), social anxiety (Cole & Hooley, 2013; Gentile et al., 2011), negative self-esteem (Ko et al., 2005), and self-reported grades (Gentile et al., 2011) in those with high scores on PVG scales and those with low scores. Looking at the extreme cases is relevant as the relationship between gaming and psychosocial problems may be curvilinear in nature, with the extreme cases suffering impairment (Allahverdi-pour, Bazargan, Farhadinasab & Moeini, 2010; van Rooij et al., 2011). This provides the following hypotheses:

Hypothesis (3): Adolescents that play online games have decreased psychosocial wellbeing in comparison with those that do not play online games.

Hypothesis (4): Problematic gamers demonstrate decreased psychosocial wellbeing more often than non-problematic gamers.

### Co-occurrence ofrisky behaviors: drinking, smoking, and cannabis use

Adolescence is an experimental period with regards to both substances and risky behaviors such as gambling (Volberg, Gupta, Griffiths, Olason & Delfabbro, 2010; Winters & Anderson, 2000). PVG can be viewed as a risky behavior as it encompasses and is associated with various problems (Rehbein et al., 2010; Sublette & Mullan, 2012). If we accept the premise that certain persons might have a genetic and/or psychological predisposition towards addictive/problematic use this might manifest itself in increases of both PVG and substance use. For instance, similar neurocognitive deficits are suspected to exist for both problem gambling and substance use (Goudriaan, Oosterlaan, de Beurs & van den Brink, 2006). Firstly, neurocognitive similarities with substance use are found for PVG as well (Kuss & Griffiths, 2012a). Impulsivity, as another example, has been found to be a typical risk factor for both problem behaviors (including alcohol use) in young people (Evenden, 1999; Khurana et al., 2013) and problematic gaming (Gentile et al., 2011; Park, Kim, Bang, Yoon & Cho, 2010; van Holst et al., 2012). There are many corresponding risk factors for a variety of alcohol and other drug problems in young people (Hawkins, Catalano & Miller, 1992), among which several have been studied and found for PVG as well; e.g. school performance, social problems, conduct problems, personality type, and attention problems (Kuss & Griffiths, 2012b).

Obviously a good number of users with these risk factors might either engage in problematic gaming or use substances. However, the supposed vulnerability is likely to result in overlap as well, as we know from the literature that overlap between various addictions is quite common (Sussman, Lisha & Griffiths, 2011). Empirical findings suggest that addictive behaviors co-occur. This includes examples such as substance use and gambling (Fisoun, Floros, Siomos, Geroukalis & Navridis, 2012; Floros, Siomos,Fisoun & Geroukalis, 2013; Griffiths, 2002; Lee, Han, Kim & Renshaw, 2013; Wood et al., 2004), and problematic computer (game) use and substance use (Grüsser, Thalemann, Albrecht & Thalemann, 2005) or gambling (Wood et al., 2004). While the relationship between PVG and substance use has been studied before, results are inconclusive and originate from small samples. In fact, the German study found no significant associations (Grüsser et al., 2005). We will focus on exploring the co-occurrence of two types of risky behavior: substance use and PVG.

Hypothesis (5): Adolescents who play online games use psychoactive substances (nicotine, cannabis, alcohol) more often than those who do not play online games.

Hypothesis (6): Adolescent substance users (nicotine, cannabis, alcohol) are more likely to be problematic gamers than non-substance users.

### Present study

The present study used data from a large adolescent sample to provide information on problematic (addictive) gaming. The role of game type, psychosocial health, and substance use were explored, with the expectation that online gaming, decreased psychosocial functioning, and substance use will be related with PVG. Compared to earlier work, the current study contributes and expands upon existing work by describing the first large sample data on the relationship between substance use and PVG.

## Methods

### Participants and procedure

The study aggregates the 2009, 2010 and 2011 samples of the yearly Dutch Monitor Study ‘Internet and Youth’. This ongoing paper and pencil study uses stratified sampling to select schools for participation based upon region, urbanization, and education level in the Netherlands. In 2009, ten schools participated (4909 questionnaires were distributed), ten schools participated in 2010 (4133 distributed) and 13 schools participated in 2011 (3756 distributed). The total sample response rates were 83% (n = 4063; 2009), 91% (n = 3745; 2010), and 84% (n = 3173; 2011). Non-response was mainly attributable to entire classes dropping out due to internal scheduling problems. With these classes excluded, the average per-class response rate was 93% (2009), 93% (2010), and 92% (2011).

In the current study the samples were used in a cross-sectional manner and aggregated over three years; the longitudinally repeated cases were removed to obtain a dataset with unique individuals. For example if an individual participated again at T2, after being included at T1, this case was removed from the aggregate dataset at 2010 (and possibly 2011). Reduced in this manner, the final aggregated dataset contains 8,478 completed cases. (For further details on the procedure see: van Rooij et al., 2010, 2012, 2011).

### Measures

#### Demographic variables

Demographic variables included sex, education level (low, i.e. vocational training or high, i.e. pre-college or university training), and Dutch secondary education learning year (first, second, third, or fourth year).

#### Game use

*Use and weekly hours spent on online gaming, casual (browser) gaming, and offline gaming.* Three types of games were distinguished: (multiplayer) online games (e.g., Call of Duty, World of Warcraft), casual (browser) games (e.g., freebrowsergames.com), and finally offline games (e.g. Sims 2). The number of hours per week spent on these game types was obtained by multiplication of two questions measuring days per week of gaming (never to [almost] daily) and average hours of gaming per day on which they play (never to 9+ hours), in line with previous studies (van Rooij et al., 2010, 2011). This was also represented as a binary use or non-use of a specific game type. The vast majority of surveyed adolescents played at least some type of game (N = 6757, 80%). Playing multiple game types was common; 41% of gamers played two game types, while 22% of the gamers played all three types of games.

*Video game Addiction Test (VAT).* The 14-item VAT scale (van Rooij et al., 2012) incorporates various aspects of behavioral addiction, including: loss of control, conflict, preoccupation/salience, coping/mood modification, and withdrawal symptoms. The VAT showed excellent reliability in the current sample (Cronbach’s *a* = 0.93). Example VAT items include: ‘How often do you find it difficult to stop gaming?’ and ‘How often do you think about gaming, even when you’re not online?’ and answer options range from ‘never’ (score 0), seldom (1), sometimes (2), to ‘often’ (3) and ‘very often’ (4) on a five-point scale.

The average score on the 14 VAT items provides an indication of average severity of the problematic behavior across all the items. The average was calculated when at least two-thirds of the scale was completed, but 99% of calculated VAT scores are averaged over 13 or 14 items. In the current study, the aim was to examine the group that scored high on the VAT. In order to distinguish this group, the average scale scores are divided into two groups. The average first group score ranges from ‘never’ to ‘sometimes’, while the answers for the second group range from ‘often’ to ‘very often’. This latter group is the category that reported the highest level of PVG.

#### Psychoactive substance use/non-use

Drinking alcohol, smoking cigarettes, and cannabis use were recoded into use or no use, as indicated by use on either weekdays (Monday through Thursday) or weekend days (Friday through Sunday) in the last month.

#### Psychosocial variables

Measures were used to establish various aspects of psychological wellbeing, focusing on self-esteem, loneliness, depressive mood, and social anxiety. Firstly, Rosenberg’s 10-item Self-Esteem Scale (Rosenberg, 1965) was used and recoded such that higher scores indicated lower self-esteem (Cronbach’s *a* = 0.87). Answers were given on a fourpoint scale. Secondly, the UCLA 10-item Loneliness Scale (Russell, Peplau & Cutrona, 1980) was used with a five point answer scale (Cronbach’s *a* = 0.85). Thirdly, a Dutch translation of the 6-item Depressive Mood List (Engels, Finkenauer, Meeus & Deković, 2001; Kandel & Davies, 1982, 1986) was used, with a 5-point answer scale (Cronbach’s *a* = 0.81). Finally, the Revised Social Anxiety Scale for Children (La Greca & Stone, 1993) subscales Social Avoidance and Distress (a = 0.85, 6 items) and Social Avoidance and Distress in general (a = 0.81, 4 items) were used with a 5-point answer scale, ranging from ‘not at all (1)’ to ‘very much (5)’. These translations have been used in various earlier Dutch studies before (van Rooij et al., 2013, 2011). For all four scales, a higher score indicates more reported problems and average scores over all items in the scale were used in analyses.

*Self-reported educational performance*. To assess (selfreported) educational performance, students were asked the following single item question: “How are you doing at school?”, with answers ranging from ‘very bad (1)’ to ‘very good’ (7).

### Analyses

Cases of high PVG and substance use are thought to have a low prevalence in Dutch adolescents (van Rooij et al., 2011; Verdurmen et al., 2011). As the hypotheses focus on the co-occurrence of these behaviors, correlational methods were not the preferred starting point for the analyses and crosstab non-parametric testing was used where applicable. For continuous measures, compared to using independent samples t-tests, an effect size of Cohen’s *d* of >0.2 is viewed as a small effect, >0.5 as medium, and >0.8 as large (Cohen, 1992).

### Ethics

The study procedures were carried out in accordance with the Declaration of Helsinki. Given the subject matter, no ethical external approval was required under Dutch law. Both children and parents receive the opportunity to refuse participation at any time without consequences: this rarely occurred.

## Results

### Sample characteristics

The sample included students from the Dutch secondary school learning year one (43%) and year two (32%). Learning years three and four (25%) were combined because learning year four had few respondents. The age in the first year was 13.2 years on average, 14.3 in the second year, and 15.5 in the third/fourth year. The overall mean respondent age was 14.2 years (*SD* = 1.1). Boys made up 49% of the sample, and education level was divided over pre-college/university (high) training (59%) and pre-vocational training (low) levels (41%).

### Comparisons between online gamers and the rest of the sample

Table 1 provides an overview of the differences between online gamers and the rest of the sample (non-online gamers) for a number of main comparison variables. Findings showed that boys were more than 4.4 times more likely than girls to be online gamers (Relative Risk or RR). Secondly, those in lower learning years (younger students) were more likely than higher learning years to play online games (39% in the first year and 31% in the third year). While a small effect was also found for cannabis use (RR = 1.25), it does not meet the criterion for acceptable significance. Online gamers were found to score higher than non-online gamers on the PVG measure (Cohen’s *d* = 0.79). There are some weak (Cohen’s *d* < 0.20) indications that online gamers have less depressive mood and have better self-esteem than non-online gamers. Further weak effect size findings show increases in loneliness, social anxiety in new situations, and worse self-reported school performance for online gamers.

**Table 1.** Demographics and substance use for online gamers and non-online gamers (rest of sample)

Categorical data	Non-online	gamer	Online	gamer	Pearson c^2^ *(p)*	Relative risk
	%	*N*	%	*N*		
Boys	40.11	1611	59.89	2405	1911.98 (<.001)	4.42
Girls	86.44	3645	13.56	572		
First year	60.84	2167	39.16	1395	35.20 (<.001)	n/a
Second year	63.71	1698	36.29	967		
Third and fourth year	68.68	1445	31.32	659		
Low education	62.76	2144	37.24	1272	2.38 (n.s.)	(1.05)
High education	64.42	3166	35.58	1749		
Alcohol use	63.57	1040	36.43	596	0.09 (n.s.)	(1.01)
Non-alcohol use	63.98	3844	36.02	2164		
Smoking	65.38	510	34.62	270	0.76 (n.s.)	(0.96)
Non-smoking	63.80	4360	36.20	2474		
Cannabis	55.38	108	44.62	87	6.56 (<.05)	(1.25)
Non-cannabis use	64.30	4728	35.70	2625		
Scale (answer range)	M *(SD)*	*N*	M *(SD)*	*N*	t-test *(p)*	Cohen’s *D*
VAT (0-4)^b^	0.37 (0.49)	3224	0.84 (0.68)	2754	-30.05 (<.001)^a^	0.79
Depressive mood (1-5)	2.23 (0.71)	5271	2.16(0.70)	2998	4.62 (<.001)	-0.10
Loneliness (1-5)	1.57 (0.50)	5260	1.64 (0.51)	2995	-6.14 (<.001)	0.14
Social anxiety (general, 1-5)	1.67 (0.70)	5239	1.71 (0.71)	2975	-2.55 (<.05)	0.06
Social anxiety (new situations, 1-5)	2.21 (0.78)	5253	2.27 (0.78)	2986	2.99 (<.01)	0.08
Negative self-esteem (1-4)	1.74 (0.55)	5233	1.68(0.52)	2964	4.80 (<.001)^a^	-0.11
School performance (1-7)	5.40(1.20)	5223	5.15 (1.23)	2984	3.34 (<.001)	-0.21

^a^ Equal variances not assumed in t-test.

^b^ As the VAT was only filled out by gamers, the total *N* is lower for this row, comparison is limited to online gamers versus non-online gamers who play other game types.

**Table 2.** Substance use and demographics divided against Video game Addiction Test categories

	Boys						Girls					
	VAT: low^a^		VAT: high^a^		Pearson	Relative	VAT: low^a^		VAT: high^a^		Fisher’s test: exact sig *(p)^c^*	Relative risk
	%	*N*	%	*N*	*x^2^ (p)*	risk	%	*N*	%	*N*		
Boys	95.18	3434	4.82	174	-							
Girls							98.74	2355	1.26	30	-	
												
First year	95.72	1498	4.28	67	1.92 (n.s.)	n/a	98.66	1174	1.34	16	1.00 (n.s.)	n/a
Second year	94.61	1105	5.39	63			98.91	729	1.09	8		
Third and fourth year	94.97	830	5.03	44			98.69	452	1.31	6		
												
Low education	94.34	1401	5.66	84	3.82 (n.s.)	(1.33)	98.17	1021	1.83	19	0.04 *(p <* .05)	(2.23)
High education	95.76	2033	4.24	90			99.18	1333	0.82	11		
												
Online gamer	93.43	2103	6.57	148	42.38 (<.001)	3.84	94.79	437	5.21	24	0.00 *(p<.001)*	20.04
Non-online gamer	98.29	1265	1.71	22			99.74	1897	0.26	5		
												
Casual gamer	95.37	2163	4.63	105	0.66 (n.s.)	(0.88)	98.73	1555	1.27	20	1.00 (n.s.)	(1.10)
Non-casual gamer	94.76	1193	5.24	66			98.85	*111*	1.15	9		
												
Offline gamer	94.89	2395	5.11	129	1.51 (n.s.)	(1.24)	98.33	1296	1.67	22	0.04 *(p <* .05)	(2.46)
Non-offline gamer	95.88	953	4.12	41			99.32	1024	0.68	7		
												
Alcohol use	92.63	704	7.37	56	15.15 (<.001)	1.87	97.11	369	2.89	11	0.00 (<.01)	3.25
Non-alcohol use	96.06	2533	3.94	104			99.11	1885	0.89	17		
												
Smoking	92.33	313	7.67	26	7.81 (<.01)	1.78	98.30	173	1.70	3	0.45 (n.s.)	(1.55)
Non-smoking	95.70	2913	4.30	131			98.90	2074	1.10	23		
												
Cannabis	89.19	99	10.81	12	9.74 (<.01)	2.42	90.32	28	9.68	3	0.00 (<.01)	8.96
Non-cannabis use	95.54	3087	4.46	144			98.92	2208	1.08	24		
												
Scale (answer range)	M *(SD)*	*N*	M *(SD)*	*N*	*t*-test *(p)*	Cohen’s *D*	M *(SD)*	*N*	M *(SD)*	*N*	*t*-test *(p)*	Cohen’s *D*
Hours of online gaming	6.90(11.52)	3368	22.62(19.78)	170	-10.28 (<.001)^b^	0.97	0.96(3.98)	2334	13.92 (19.38)	*29*	-3.60 (<.01)^b^	0.93
Hours of casual gaming	1.84(4.19)	3356	4.16(9.80)	171	-3.08 (<.001)^b^	0.31	1.73(4.08)	2326	1.89 (2.58)	*29*	-0.21 (n.s.)	0.05
Hours of offline gaming	5.09(8.60)	3348	11.23 (15.64)	170	-5.08 (<.001)^b^	0.49	2.22(5.11)	2320	13.29(17.70)	*29*	-3.37 (<.01)^b^	0.85
												
Depressive mood (1-5)	2.03 (0.64)	3423	2.70 (0.82)	172	-10.60 (<.001)^b^	0.91	2.31 (0.72)	2345	3.24(0.79)	30	-7.05 (<.001)	1.23
Loneliness (1-5)	1.61 (0.48)	3421	1.97(0.73)	173	-6.55 (<.001)^b^	0.58	1.58 (0.51)	2342	2.06(0.71)	30	-3.63 (<.01)^b^	0.78
Social anxiety (general, 1-5)	1.65 (0.65)	3414	2.15(0.97)	171	-6.68 (<.001)^b^	0.61	1.73 (0.75)	2339	2.68(1.14)	30	-4.58 (<.001)^b^	0.98
Social anxiety (new, 1-5)	2.16(0.73)	3420	2.60 (0.94)	173	-6.05 (<.001)^b^	0.52	2.43 (0.81)	2345	2.89 (0.95)	30	-3.12 (<.01)	0.52
Negative self-esteem (1-4)	1.60(0.47)	3421	1.98(0.62)	173	-7.84 (<.001)^b^	0.69	1.81 (0.58)	2343	2.34 (0.72)	30	-4.96 (<.001)	0.81
School performance (1-7)	5.20(1.21)	3383	4.69(1.46)	171	4.43 (<.001)^b^	-0.38	5.52(1.12)	2319	4.63 (1.45)	30	3.33 (<.01)^b^	-0.69

^a^ The VAT was only filled out by gamers (of any type of game), which means the current table contains data on a self-reported gamer subsample.

^b^ Equal variances not assumed in *t*-test.

^c^ Expected counts were lower than five in some cells: Fisher’s exact test was employed in this section.

### Problematic gaming and its hypothesized associates

Gender plays a crucial role in gaming: generally boys game for longer periods of time and more frequently. The findings from Table 1 demonstrate this holds true for online gaming, which is strongly associated with both PVG and gender. Therefore, the findings in Table 2 were split by gender. As Table 2 contains those respondents that filled out the VAT list, which could be skipped by non-gamers, the table contains results for a gamer subsample.

For boys, gamers that play online games were almost four times more likely to score high on PVG than non-online gamers (RR = 3.84). No differences were found for casual and offline game types. For all three types of substance use, differences were found: those who drink alcohol (RR =1.9), smoke cigarettes (RR = 1.8), or use cannabis (RR = 2.4) were approximately two times more likely to score high on PVG. On continuous measures, the high problematic gamer group was found to spend much more time playing online games (large effect, Cohen’s *d* = 0.97), more time playing offline games (medium effect, Cohen’s *d* = 0.49), and more time playing on casual games (small effect, Cohen’s *d* = 0.31). The time spent on online games was a lot higher on average as well, with 23 hours for high problematic gamers, versus 11 hours spent on offline games and 4 hours on casual games. The male high problematic gamer group was also found to score lower on psychosocial wellbeing; a large effect was found for increased depressive mood, medium effects were found for loneliness, social anxiety (generalized and new situations), negative self-esteem, and a low effect for lower school performance.

Among girls, the high problematic group was smaller compared to males at 1.3% of female gamers (compared to 4.8% of boys scored high on PVG). Consequently, the absolute numbers in Table 2 for girls were low, with a maximum of 30 in the problematic group. This warrants caution with the interpretation of cross-tab Chi-square testing, where some observed cells contain less than 10 cases and some expected cell counts were lower than five. Nonetheless - and similar to the boys - online gaming girls seem more likely to score high on PVG (RR = 20.0). Female cannabis users (RR = 3.3) and alcohol drinkers (RR = 9.0) also seemed more likely to be problematic gamers. The time spent on both online and offline games was found to be higher in the problematic group of female gamers, with a strong effect size. However, the average weekly time spent on these games by girls seemed lower for online games, with an average of 14 hours per week. Again, the high problematic group scored worse on all indicators of psychosocial wellbeing: strong effects were found for depressive mood and general social anxiety, and medium effects for loneliness, negative self-esteem, social anxiety in new situations, and decreased school performance.

## Discussion

The current study used aggregated large sample data (*N* = 8,478) to study problematic (addictive) gaming in an adolescent age group. Findings confirmed that problematic gaming is most common amongst adolescent gamers that play multiplayer online games. Gamers that played online games were almost four times more likely to score high on a measure of PVG. Gender played a large role in both game preference and PVG: boys (60%) were more likely to play online games than girls (14%) and problematic gamers were more likely to be boys (5%) than girls (1%). While problematic gamers spent more time on all three types of gaming, online gaming showed both the highest average number of hours (23 hours per week) and the highest effect size increase (Cohen’s *d* = 0.97) for the high problematic male gamers.

Beyond being male, slightly younger, and more prone to PVG, no large differences were found between online multiplayer gamers and the rest of the sample. For instance, no increases in psychoactive substance use and no (substantial) increases in psychosocial problems were found. However, high PVG was associated with higher substance use and psychosocial problems for both boys and girls. Nicotine, alcohol, and cannabis using boys were almost twice more likely to report high PVG. The high PVG group of girls was very small in an absolute sense (n = 30). Consequently, there were some indications that psychoactive substance-using girls, specifically alcohol and cannabis, scored high on PVG more often, but caution is warranted with interpretation due to small group size. There is a need to better understand this group, and future research may wish to explore PVG in girls. High problematic gamers - both boys and girls - showed increases on depressive mood (large effect), loneliness, social anxiety (both generalized and new situations), negative self-esteem, and self-reported lower school performance.

The present findings align with previous literature and expand it through exploration of the association between substance use and PVG. The role of online multiplayer games found here is supported by other studies (Council on Science and Public Health, 2007; Rehbein et al., 2010; van Rooij et al., 2010). Together, these findings suggest that future research should examine specific mechanisms and characteristics present in these online games and that increase their potential to be addictive. The DSM-5 focuses exclusively on internet (online) gaming. Focusing on one specific game type seems premature given the data presented here. While online games are indeed found to be most problematic, playing multiple game types was common (63% of gamers play two or more game types). Playing online may facilitate the problematic behavior (Griffiths, King & Demetrovics, 2014).

While few studies have been done on the specific link between PVG and psychoactive substance use, the findings fit with results in the related area of gambling. Griffiths and Sutherland found that adolescent gamblers (11-16 years of age) were more likely to drink alcohol, smoke cigarettes, and take illicit drugs (Griffiths, Parke & Wood, 2002; Griffiths & Sutherland, 1998). The current study found high PVG and substance use were likely to co-occur, unlike earlier German work (Grüsser et al., 2005). Our findings may provide some support for the idea of an underlying vulnerability to ‘addictive’ behaviors that predisposes adolescents to both forms of risky behavior (Hawkins et al., 1992; Shaffer et al., 2004).

A relationship was found between decreased psychosocial wellbeing, low school performance, and high PVG. Depressive mood, social anxiety, negative self-esteem, loneliness, and school performance were all worse in the high PVG group - for both boys and girls. While these findings fit with the literature, they provide an indication that the group of high problematic gamers reports problems beyond the PVG behavior itself. As the current study uses a cross-sectional approach by design, it cannot be known if these decreases are a cause or a result of PVG. The literature suggests it might be both. Firstly, there is some evidence that children with more psychosocial problems, such as social anxiety, prefer online social interactions (Valkenburg & Peter, 2011). Secondly, a two-wave panel study among 543 Dutch gamers (Lemmens, Valkenburg & Peter, 2011) showed that social competence, self-esteem, and loneliness were predictors of changes in PVG (with low effect sizes), while loneliness was also a consequence. Another study showed that greater amounts of gaming, lower social competence, and greater impulsivity were risk factors for PVG while depression, anxiety, social phobia, and lower school performance seem to act as outcomes (Gentile et al., 2011).

The use of a large, aggregated sample is a strength of this study. However, the study also has some limitations. Firstly, the types of gaming were divided into three broad categories. This makes practical sense as earlier research has also used this distinction (van Rooij et al., 2010), but it loses details like game type (Elliott, Golub, Ream & Dunlap, 2012; Ghuman & Griffiths, 2012). Subdividing the scale for PVG provides information to distinguish low and high problematic groups but the cut-off score, whilst justified, could be debated (van Rooij et al., 2011). Also, given the low prevalence of some events we considered, we could not adjust analyses for potential clustering effects (either by class or year). Finally, the data in this study were exclusively self-reported. Future research might benefit from incorporating externally valid outcome measures, such as grades or software-based behavioral tracking (Griffiths & Whitty, 2010).

In conclusion, the present study used a large sample to expand the research findings of three potential characteristics of problematic (addictive) gaming: the role of game type, substance use, and psychosocial health. Findings revealed a mixed picture. It appears that online gaming in general is not necessarily associated with problems. In fact, there are some weak indications that it is associated with decreased depressive mood and improved self-esteem. However, online games are most often implicated in problematic use as well, and problematic users show decreased psychosocial functioning and lower grades. Moreover, associations with alcohol, nicotine, and cannabis use are found for boys. The findings presented here encourage further exploration of the role of psychoactive substance use in PVG and show that it might be premature to ignore the role of non-internet games in the study of ‘Internet Gaming Disorder’.
